# Wing Interferential Patterns (WIPs) and machine learning for the classification of some *Aedes* species of medical interest

**DOI:** 10.1038/s41598-023-44945-3

**Published:** 2023-10-17

**Authors:** Arnaud Cannet, Camille Simon-Chane, Aymeric Histace, Mohammad Akhoundi, Olivier Romain, Marc Souchaud, Pierre Jacob, Darian Sereno, Louis-Clément Gouagna, Philippe Bousses, Françoise Mathieu-Daude, Denis Sereno

**Affiliations:** 1 Direction des affaires sanitaires et sociales de la Nouvelle-Calédonie, Nouméa, France; 2 ETIS UMR 8051, Cergy Paris University, ENSEA, CNRS, 95000 Cergy, France; 3 Parasitology-Mycology, Hopital Avicenne, AP-HP, Bobigny, France; 4 Cergy Paris University, Cergy, France; 5 Univ. Bordeaux, CNRS, Bordeaux INP, LaBRI, UMR 5800, 33400 Talence, France; 6 InterTryp, Univ Montpellier, IRD-CIRAD, Infectiology Medical Entomology and One Health Research Group, Montpellier, France; 7 MIVEGEC, Univ Montpellier, CNRS, IRD, Montpellier, France; 8 Institut Louis Malardé, Tahiti, French Polynesia

**Keywords:** Zoology, Entomology, Infectious diseases

## Abstract

Hematophagous insects belonging to the *Aedes* genus are proven vectors of viral and filarial pathogens of medical interest. *Aedes albopictus* is an increasingly important vector because of its rapid worldwide expansion. In the context of global climate change and the emergence of zoonotic infectious diseases, identification tools with field application are required to strengthen efforts in the entomological survey of arthropods with medical interest. Large scales and proactive entomological surveys of *Aedes* mosquitoes need skilled technicians and/or costly technical equipment, further puzzled by the vast amount of named species. In this study, we developed an automatic classification system of *Aedes* species by taking advantage of the species-specific marker displayed by Wing Interferential Patterns. A database holding 494 photomicrographs of 24 *Aedes* spp. from which those documented with more than ten pictures have undergone a deep learning methodology to train a convolutional neural network and test its accuracy to classify samples at the genus, subgenus, and species taxonomic levels. We recorded an accuracy of 95% at the genus level and > 85% for two (*Ochlerotatus* and *Stegomyia*) out of three subgenera tested. Lastly, eight were accurately classified among the 10 *Aedes* sp. that have undergone a training process with an overall accuracy of > 70%. Altogether, these results demonstrate the potential of this methodology for *Aedes* species identification and will represent a tool for the future implementation of large-scale entomological surveys.

## Introduction

Pathogens (viruses, bacteria, parasites) transmitted by arthropods are among the most devastating infectious agents that scourge the human population worldwide. Hematophagous insects belonging to the *Aedes* genus are proven vectors of viral (chikungunya, Zika, dengue, Rift Valley fever, etc.) and filarial pathogens (*Brugia malayi*, *Wuchereria bancrofti*) of medical interest. The *Aedes* genus encompasses 79 subgenera, with more than 900 valid named species and 63 subspecies (https://www.itis.gov/). In 2000, a revision of the *Aedes* taxonomy was proposed, and the subgenus *Ochlerotatus* was “raised” to the level of the genus^1^. Nevertheless, this remains a matter of debate^2^, and a stable classification that considers utility and the current knowledge of the evolutionary relationship was proposed^3^. Still, much of the scientific literature refers to the subgenus *Ochlerotatus*. Out of 79 described subgenera, 15 contain named species with medical interests in transmitting viruses or parasites. Most *Aedes* species with medical interest fall into the *Ochlerotatus* (Lynch Arribalzaga, 1891), *Stegomyia* (Theobald, 1901), *Aedimorphus* (Meigen, 1818), or *Finlaya* (Theobald, 1903) subgenera. Among species with medical interest, only 2 disclose a worldwide distribution in 6 out of 7 continents: *Ae. aegypti* (Linnaeus 1762) and *Ae. albopictus* (Skuse, 1895). *Aedes albopictus,* a vector of public health importance, has encompassed a rapid change in its global distribution due to the worldwide trade in second-hand tires, often containing stagnant water, making it an ideal place for eggs and larvae, and its easiness to adapt to new environments, even in a temperate climate. This expansion offers opportunities for arboviruses (viruses transmitted by arthropods) to circulate in new areas, becoming a common cause of epidemics in *Ae. aegypti*-free countries. In addition to the mentioned species, at least seven others are indigenous mosquitoes on more than two continents (https://www.worldatlas.com/continents accessed on 01/24/2023).

For the entomological survey, identifying potential vectors belonging to the *Aedes* genus is typically based on intrinsic and extrinsic morphological criteria with morphological keys of determination as a tool. This is a time-consuming task that requires expertise and training. The discriminative morphological identification of adult *Ae. albopictus* is readily amenable by looking at bold black shiny scales and distinct silver-white scales on the palpus and tarsi^4^. Additionally, the dorsal scutum is black, with a distinguishing white stripe in the center, beginning at the dorsal surface of the head and continuing along the thorax. This character is nevertheless present in the specimen of the Scutellaris groups belonging to the *Stegomyia* subgenus, roughly 40 species. It cannot as perse be considered a diagnostic character. It is a medium-sized mosquito (2.0–10.0 mm; males are on average 20% smaller than females). Differential morphology of males from females includes the plumose antennae and modified mouthparts for nectar feeding. Dark scales cover the abdominal tergites. Legs are black with white basal scales on each tarsal segment. The abdomen narrows into a point characteristic of the genus *Aedes*. Adult *Ae. aegypti* can be recognized by white marks on the legs and a marking lyre on the thorax. In areas with multiple sympatric *Aedes* species with or without medical importance, these distinctions according to morphological characteristics are only sometimes easily discriminative. In addition, it is common for older adult specimens to have missed or damaged body parts or characters (e.g., scales, legs) that are essential for accurate identifications. Specimens with damage in critical regions for diagnostic characters separating vector species from closely related non-vector ones further puzzled entomological surveys.

Machine learning models, especially convolutional neural networks, can classify objects by identifying visible and non-visible features to the naked eye^5^. They are of choice and have been extensively used for insect identification involving whole insect image recognition. They demonstrate astonishing accuracy on a wide range of Arthropods^6–8^, including Culicidae^9–11^. Features related to animal behavior (e.g., flying and walking trajectories, postures, etc.)^12^, allowing at-distance identification of alive specimens, have also been tested. Models based on insect morphology imaging of immobilized insects, an approach close to the entomological expertise deployed to identify insects that have inter-genus inter-species high morphological similarities, require a considerable number of data for training each Genus/species to learn the features and gain validation accuracy^10,13,14^. Databases needed to train such models on whole insect recognition are filled with pictures of several poses, dorsal–ventral, etc., to collect taxonomic discrimant characters^6,15–17^.

Wing Interference Patterns (WIPs) have received attention for their taxonomic potential^18–20^. The thin-film interference occurring on the wings’ transparent membrane allows the formation of a colored pattern. These WIPs significantly vary among specimens belonging to different species but moderately between representatives of the same species or between sexes. Unlike the angle-dependent iridescence effect of a flat film, the newton color series displayed is proportional to the thickness of the wing membrane at any given point, wing structures acting as diopters ensuring the WIPs appear essentially non-iridescent^19^. In previous papers, we have shown the value of WIPs for Glossina and Anopheles classification^21,22^.

In the context of global climate change and the emergence of zoonotic infectious diseases, identification tools with field application are required to strengthen efforts in the entomological survey of arthropods with medical interest^23,24^. Therefore, implementing new and affordable methods to accurately identify mosquitoes of the Aedes genus is a prerequisite for an entomological survey. To this aim, we have explored the accuracy and reliability of Wing Interferential Patterns (WIPs) to accurately identify *Aedes* specimens and classify them using a deep learning (DL) procedure.

## Material and methods

### *Aedes* selection and storage

**Figure 1:**
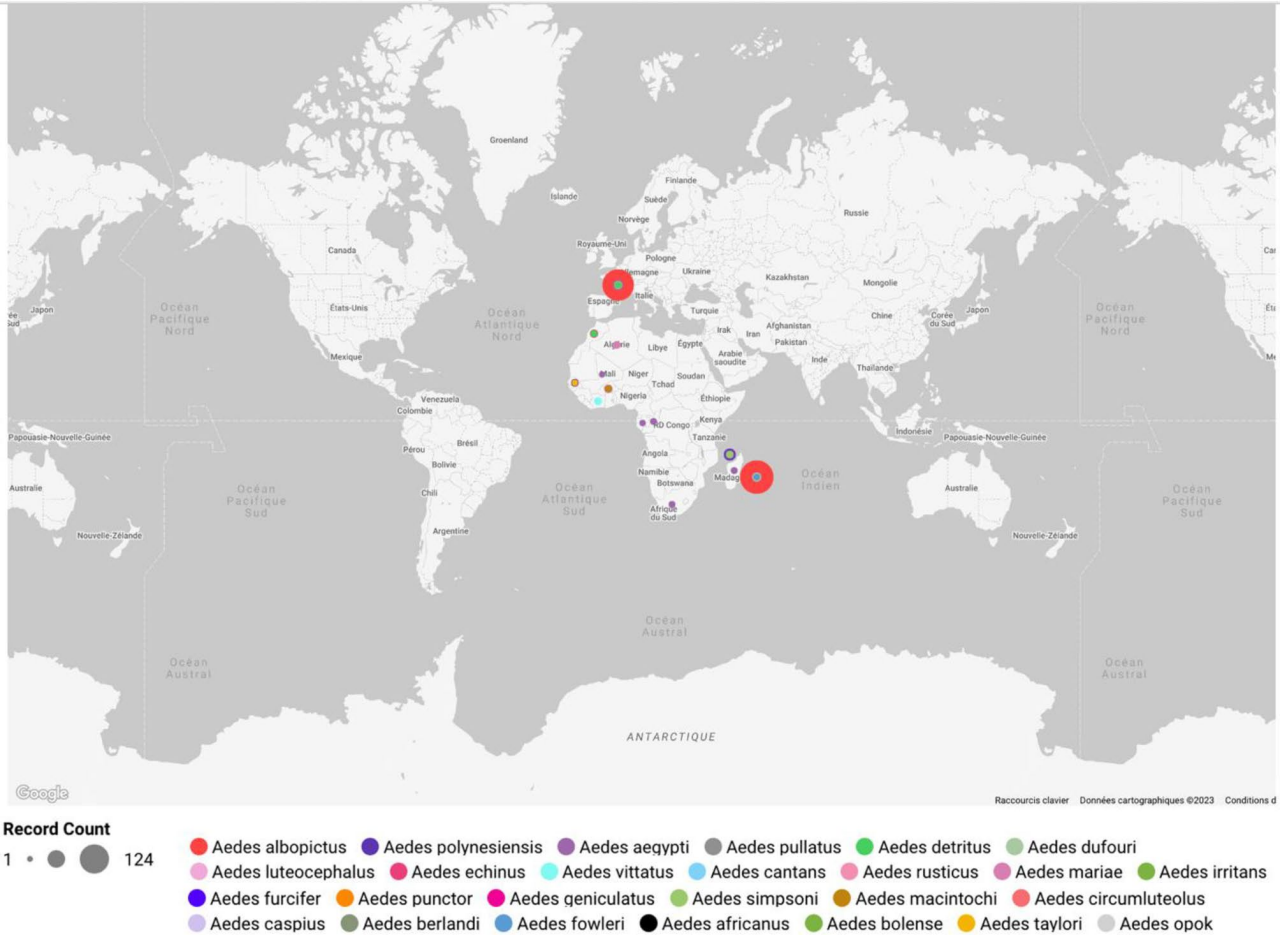
Geographic distribution of samples included in the study using Google Looker Studio (https://lookerstudio.google.com/overview).

The first WIPs reference collection of Culicidae gathers samples belonging to the *Aedes* genus using well-established *Aedes albopictus* laboratory breeds. Specimens were also selected in the ARIM collection (https://arim.ird.fr/) of IRD (Institut de Recherche pour le Développement). In addition, samples collected *in natura* whose identification was performed by trained entomologists at the time of their catch with available regional identification keys were also included in the database. The description of the samples used in this study is given in Table 1, and the worldwide distribution of samples included in the study is illustrated in Fig. 1. A complete list of the Diptera dataset used in this study and of species included in the database with their associated class is available in the supplementary data and the figshare server (Supdata: ID of species included in the study).

**Table 1:** List of named *Aedes* species and description of samples included in the dataset (A) and of Aedes species having a medical interest but not included in our dataset (B).

**A** *Aedes* spp. in our database	Med int*	Or	Year	N	Country code	Morphological identification by trained entomologists
*Aedes (Stegomyia) albopictus*	Yes	C-W	2015	**239**	250, 638	Ph Bousses
*Aedes (Stegomyia) aegypti*	Yes	W	2010	**55**	854, 266, 710	ND, Ph Bousses
*Aedes (Stegomyia) polynesiensis*	Yes	W	2016	**27**	876	F Mathieu-Daudé
*Aedes (Stegomyia) africanus*	Yes	W	2010	**2**	ND	JP Hervy
*Aedes (Stegomyia) opok*	No	W	ND	**2**	ND	JP Hervy
*Aedes (Stegomyia) luteocephalus*	Yes	W	1967	**13**	854	G Pichon, F Rodhain
*Aedes (Stegomyia) simpsoni*	No	W	ND	**7**	175	JP Hervy, ND
*Aedes (Ochlerotatus) detritus*	No	W	ND	**22**	504, 250	H Bailly-Choumara
*Aedes (Ochlerotatus) mariae*	No	W	1929	**13**	12	ND
*Aedes (Ochlerotatus) pullatus*	No	W	ND	**15**	ND	ND
*Aedes (Ochlerotatus) punctor*	No	W	1967	**12**	250	ND
*Aedes (Ochlerotatus) rusticus*	No	W	1967	**8**	250	ND
*Aedes (Ocherotatus) dufouri*	No	W	2012	**14**	686	Ph Bousses
*Aedes (Ochlerotatus) cantans*	No	W	1960	**9**	250	H Bailly-Choumara
*Aedes (Neomelaniconion) bolense*	No	W	1950	**2**	ND	ND
*Aedes (Neomelaniconion) macintoshi*	Yes	W	1950	**5**	854	J Hamon
*Aedes (Neomelaniconion) circumluteolus*	No	W	1955	**5**	ND	J Hamon
*Aedes (Aedimorphus) fowleri*	No	W	2012	**3**	638	Ph Bousses
*Aedes (Aedimorphus) irritans*	No	W	1986	**7**	686	JP Hervy
*Aedes (Diceromyia) furcifer*	Yes	W	1986	**7**	686	JP Hervy
*Aedes (Diceromyia) taylori*	Yes	W	1987	**2**	686	J Hamon
*Aedes (Finlaya) geniculatus*	No	W	2015	**6**	ND	G Le Goff
*Aedes (Finlaya) echinus*	No	W	1966	**10**	504	H Bailly-Choumara
*Aedes (Fredwardsius) vittatus*	Yes	W	1960	**9**	384	J Hamon
**B** *Aedes* spp*.* of medical interest not in our database						
*Aedes (Aedimorphus) cumminsii*						
*Aedes (Aedimorphus) dalzieli*						
*Aedes (Aedimorphus) dentatus*						
*Aedes (Aedimorphus) hirsutus*						
*Aedes (Aedimorphus) ochraceus*						
*Aedes (Aedimorphus) vexans*						
*Aedes (Catageionyia) minutus*						
*Aedes (Catageiomyia) argenteopunctatus*						
*Aedes (Catageiomyia) tarsalis*						
*Aedes (Downsiomyia) harinasutai*						
*Aedes (Downsiomyia) niveus*						
*Aedes (Elpeytonius) simulans*						
*Aedes (Finlaya) fijiensis*						
*Aedes (Finlaya) kochi*						
*Aedes (Finlaya) poicilius*						
*Aedes (Georgecraigius) atropalpus*						
*Aedes (Georgecraigius) epactius*						
*Aedes (Hulecoeteomyia) japonicus*						
*Aedes (Hulecoeteomyia) koreicus*						
*Aedes (Neomelaniconion) palpalis*						
*Aedes (Ochlerotatus) angustivittatus*						
*Aedes (Ochlerotatus) atlanticus*						
*Aedes ((Ochlerotatus) canadanensis*						
*Aedes (Ochlerotatus) cantator*						
*Aedes (Ochlerotatus) condolescens*						
*Aedes (Ochlerotatus) dorsalis*						
*Aedes (Ochlerotatus) dupreei*						
*Aedes (Ochlerotatus) excrucians*						
*Aedes (Ochlerotatus) fitchii*						
*Aedes (Ochlerotatus) grossbecki*						
*Aedes (Ochlerotatus) infirmatus*						
*Aedes (Ochlerotatus) melanimon*						
*Aedes (Ochlerotatus) nigromaculis*						
*Aedes (Ochlerotatus) provocans*						
*Aedes (Ochlerotatus) scapularis*						
*Aedes (Ochlerotatus) sollicitans*						
*Aedes (Ochlerotatus) squamiger*						
*Aedes (Ochlerotatus) sticticus*						
*Aedes (Ochlerotatus) stimulans*						
*Aedes (Ochlerotatus) taeniorhynchus*						
*Aedes (Ochlerotatus) trivittatus*						
*Aedes (Ochlerotatus) vigilax*						
*Aedes (Polyleptomyia) albocephalus*						
*Aedes (Protomacleaya) triseratus*						
*Aedes (Rampamyia) notoscriptus*						
*Aedes (Stegomyia) scutellaris*						
*Aedes (Tanakaius) togoi*						

*Medical interest according to the WRBU database (https://wrbu.si.edu/vectorspecies?field_family_target_id=1194&title=&field_mt_products_tags_target_id=&field_pathogens_target_id=&field_geographic_locations_target_id=&items_per_page=30) and Wilkerson et al.^3^.

& ISO 3166–1 country code available at (https://www.atlas-monde.net/codes-iso/).

Or, the sample’s origin; W, wild; C, colony; N, number of samples processed.

### Image acquisition and database construction

The procedures applied to capture WIPs were the same as those described for *Glossina* sp. WIPs acquisition^21^. Wings were dissected and deposited on a glass slide, adding a small cover slide. The picture was taken with a Keyence™ VHX 1000 microscope, using the VH-Z20r camera and a Keyence VH K20 adapter, allowing an illumination incidence of 10°. The High Dynamic Range (HDR) function was used for all photos. All pictures were enlarged to a maximal occupancy, making the size of the wing not a discriminating parameter for insect species identification. The numerical parameters settled are the same as described and detailed in the publication on *Glossina* spp.^21^.

### Collected dataset, image pre-processing, and dataset splitting for training/learning and validation

A rich annotated image dataset, including 494 pictures of 24 *Aedes* species, is available (figshare: https://figshare.com/s/597f4e2f2d8206e09603). Under-sampled *Aedes* species (less than ten samples/pictures) were discarded for training to prevent overfitting. All processed images were resized to 256 and 116 pixels for width and height, respectively. Pixel values were normalized within the (0, 1) range.

**Figure 2:**
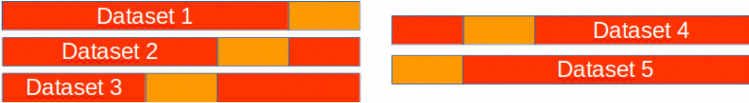
Schematic representation of the dataset splitting for learning (red) and testing (orange) from Cannet et al.^21^.

The dataset was then prepared for k-fold cross-validation, with k = 5 (Fig. 1). K-fold cross-validation is a classic approach to evaluate the robustness of a machine learning method, including Deep Learning ones. Mainly, for this study, the dataset was randomly shuffled and partitioned into k equal-size subsets with similar class distributions. A separately learned classifier was evaluated for each subgroup using kth of the whole dataset for validation and the remaining k−1 as training data (see Fig. 2 for illustration). This strategy allowed for measuring the mean accuracy of 5 distinct models and is the most accurate neural network performance estimation method^25^.

**Figure 3:**
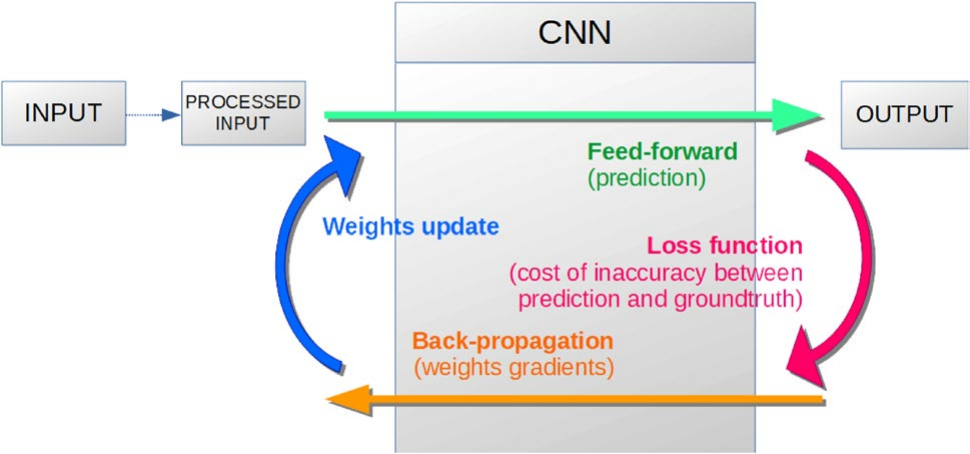
Schematic representation of the pipeline process developed for *Glossina* identification using the Convolutional Neural Network (CNN) approach from Cannet et al.^21^.

This strategy allowed for measuring the mean accuracy of the five distinct classifiers. Among all existing machine learning methods, Deep Convolutional Neural Networks and their different architectures have shown in the last decade to be the most adapted for image classification. Compared to classic shallow methods (Support Vector Machine, Random Forest, and Boosting-based approaches for the main ones), they do not need hand-crafted features as input of the learning process: the selection of the best features is intrinsic to the method itself and is particularly well adapted to the WIPs scenario. A pipeline overview of the complete training procedure using CNN is shown in Fig. 3.

### Training of the neural network (CNN)

The data used to develop the Deep Learning model includes Culicidae and non-Culicidae specimens^26^. The methodology used was initially developed and tested on Tetse flies^21^. Briefly, the original CNN architecture MobileNet^27^, ResNet^28^, and YOLOv2^29^ architecture were deemed for automatically classifying *Aedes* sp. with the abovementioned dataset. Compared to classic Deep Learning, ours is more compact to cope with the specificity of our dataset in terms of size; therefore, thinner image recognition and classification architecture were developed to consider its reduced size. The first one is inspired by MobileNet, which takes advantage of depth-wise convolution^27^. We propose to work with only one depth-wise convolution per layer of the CNN architecture to reduce the complexity and the number of extracted features. In addition, batch normalization was set to speed up and stabilize the training process^30^.

In addition to this first compact CNN architecture based on MobileNet, two interconnected layers like VGG^31^ for YOLOv2 were applied with a DarkNet-19 architecture^29^, as performed on tsetse flies samples^21^. As this architecture tends to over-fit the training set (meaning a lack of generalization of the performance when data other than the training data set are considered), we test two reduced architectures, i.e., using 1 or 2 scales less than the original network. For clarity, we called them DarkNet-9 (8 convolution layers and 1 classification layer) and DarkNet-14 (13 convolution layers and 1 classification layer). We also reproduced the ResNet18 architecture from He and collaborator^28^ and trained it from random initialization. Even if this architecture seems too “deep” (may lead to overfitting) compared to our other architectures, the intrinsic properties of ResNet18, residual connections, allow convergence of the training procedure^21^.

We used a standard approach (shallow approach) based on extracting SURF descriptors (an efficient implementation of the classic SIFT descriptors), a Bag of Features (BoF) representation using a 4000 codewords dictionary, and an SVM with a standard polynomial kernel similar to it was proposed in Sereno et al.^32^. For each task, we only use one fully connected layer with the softmax activation to predict the probability that an image belongs to the correct class. We train our networks using Stochastic Gradient Descent (SGD) with a learning rate of 10^2^ and a momentum of 0:9 for 30 epochs. The method was developed on a workstation with a quad-core CPU 3.0 GHz and 16Go RAM. Information on the training options, accuracy, and sensitivity, as well as the code, are available at https://github.com/marcensea/diptera-wips/commit/12f39ab500a3f820cfb817c67ef25c580942301d.

## Results

### Training and classification

We explored the training classifier accuracy on the *Aedes* dataset and on datasets of Culicidae that do not belong to the *Aedes* genus (non-*Aedes)* and from mosquitoes that do not belong to the Culicidae family (non-Culicidae) as negative samples. We trained the CNN on such a combination to improve the model’s accuracy. The database initially filled with a total of 494 pictures of *Aedes* spp. WIPs pictures. Since we cannot fill the database with all *Aedes* species acting as primary vectors of viruses, parasites, or bacteria with a medical interest, we first focused on a restricted set of species (Table 1). Using this pictures-set, we ascertained the accuracy of the process to discriminate the *Aedes* genus (Meigen, 1818) from other Culicidae (Meigen, 1818), including Anophelinae (Grassi, 1900), Culicinae (Meigen, 1818), and non-Culicidae samples members (e.g., Psychodidae, Glossinidae…). From our dataset and process, the automatic classification process accuracy is rather good and higher than 95% (Table 2).

**Table 2:** Test for accuracy of the DL (Deep Learning) process for the *Aedes* (Meigen, 1818) genus identification.

		Predicted		
		*Aedes* spp.	Other Culicidae	Non Culicidae
Truth	*Aedes* spp. N = 84	80 (**95.2%)**	4	0
	Other Culicidae N = 259	2	256 (**98.8%**)	1
	Non Culicidae N = 675	0	1	674 (**99.9%**)

N, number of pictures in the test subset. Accuracy data (%) in bold.

We also evaluated the DL process's accuracy at the subgenus level (Table 3). The *Aedes* genus encompasses 79 subgenera, with only 14 gathering species documented to act as primary vectors of viruses, parasites, and bacteria of medical interest. As mentioned in the introductory section, the subgenus *Ochlerotatus* was “raised” to the level of the genus in 2000^1^. Considering that a large number of the scientific literature refers to the subgenus *Ochlerotatus*, we refer to this taxonomic nomenclature to carry out our analysis^33^. Our *Aedes* dataset does not represent the overall diversity at the subgenera level. Nevertheless, the computed accuracy for the identification process is high (> 85%) for the *Stegomyia* and *Ochlerotatus* subgenera, with a classification accuracy of 97.0 and 86.7%, respectively. Still, it fails to address the *Finlaya* subgenus accurately. Such low accuracy might be due to the small sample size available to train and test the process for *Finlaya*. Therefore, efforts in implementing the database with enough specimens representative of the *Finlaya* and other subgenera must be made to address our methodology’s accuracy in addressing the classification process at the subgenus taxonomic level. Overall, our methods are the first based on deep learning with the versatility of automatically handling samples at the subgenus level, which might be of interest to speed and facilitate the identification of specimens of *Aedes* species of medical interest.

**Table 3:**
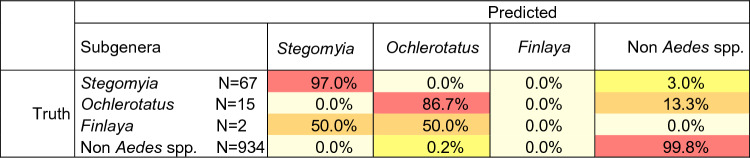
Test for accuracy (%) of the DL classification process at the subgenus taxonomic level.

N, number of pictures in the test subset, Accuracy in %.

Finally, the reliability of the DL model to accurately classify WIPs pictures of 10 *Aedes* species was calculated, and the results are presented in Table 4. Variable level of accuracy is recorded, ranging from not accurate (0.00%) to perfect classification (100.00%). A perfect accuracy (100.00%) is achieved for 50% of *Aedes* species whose WIPs pictures are filled in the dataset. More than 75% accuracy in classification is recorded for 7 *Aedes* species, but the DL methods failed to assign 2 *Aedes* species correctly (*Ae. echinus* and *Ae. mariae*) (Table 4). Even if populations of *Ae. albopictus* bear a high variability independently of whether the populations are native or invasive^34,35^, we record a high classification accuracy (Table 4). The DL approach based on WIPs can manage the diversity of specimens sampled in two distant geographic areas, France and La Réunion. *Aedes aegypti* exists in two subspecies or forms: the domestic ecotype, *Ae. aegypti aegypti* found outside Africa and the presumed ancestral *Ae. aegypti formosus* occurring in sub-Saharan Africa in sympatry with *Ae. aegypti aegypti* in some ecologies^36,37^. Our samples of *Ae. aegypti* originating from sub-Saharan Africa were not identified at the subspecies level. They may be a mix of the two ecotypes with subtle variations in their WIPs. This might be the underlying factor triggering the lower accuracy (72.7%) of the identification process of *Ae. aegypti* in our dataset, as compared to the accuracy recorded for *Ae. albopictus* (95.8%) classification. This deserves to be investigated. Nevertheless, the overall calculated specific recognition of samples belonging to *Aedes* remains satisfactory.

**Table 4:**
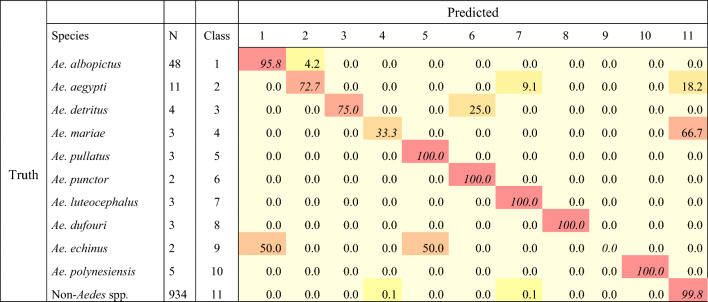
Accuracy in % of the DL process for *Aedes* spp. classification.

Significant values are in [italics].

N, number of images in the test subset.

#### Misclassified pictures

**Figure 4:**
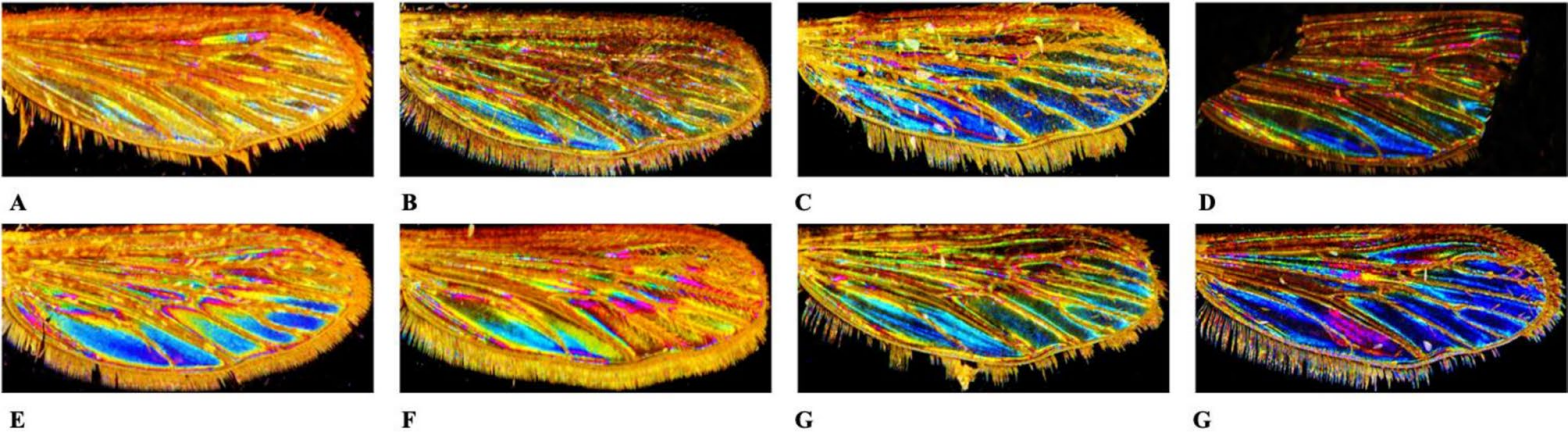
Selected examples of misclassified pictures. *Ae. albopictus* misidentified as *Ae. aegypti* (**A**), *Ae. albopictus* misidentified as *Ae. aegypti* (**B**), *Ae. aegypti* misidentified as *Ae. luteocephalus* (**C**), *Ae. aegypti* misidentified as *Ae. albopictus* (**D**), *Ae. mariae* misidentified as *Cx. neavei* (**E**), *Ae. echinus* misidentified as *Ae. pullatus* (**F**), *Ae. polynesiensis* misidentified as *Ae. albopictus* (**G**), *Ae. polynesiensis* misidentified as *Cx. neavei* (**H**).

Inspection of the machine learning model weaknesses helps identify underlying problems. This can be performed via a review of the miss-predicted images. This will provide insights into what makes a photo hard to classify for the model. Our data set takes advantage of having to be filled with pictures of samples collected *in natura* or from laboratory colonies but also preserved for an extended period or damaged. This large panel of images taken with the same microscope under the same light condition, preparation process, and preprocessing methodologies will help to delineate factors (e.g., age, sample origin, sample preservation…) affecting the classification process. In Fig. 4, selected examples are presented. Deep learning models rely more on textures than shapes. A more extensive training set can avoid pitfalls linked to photo or sample quality, prevent confusing set-ups when taking pictures, and improve the accuracy of the automated classification. A guideline can be added to the application to advise participants to make good images of Culicidae samples.

**Figure 5:**
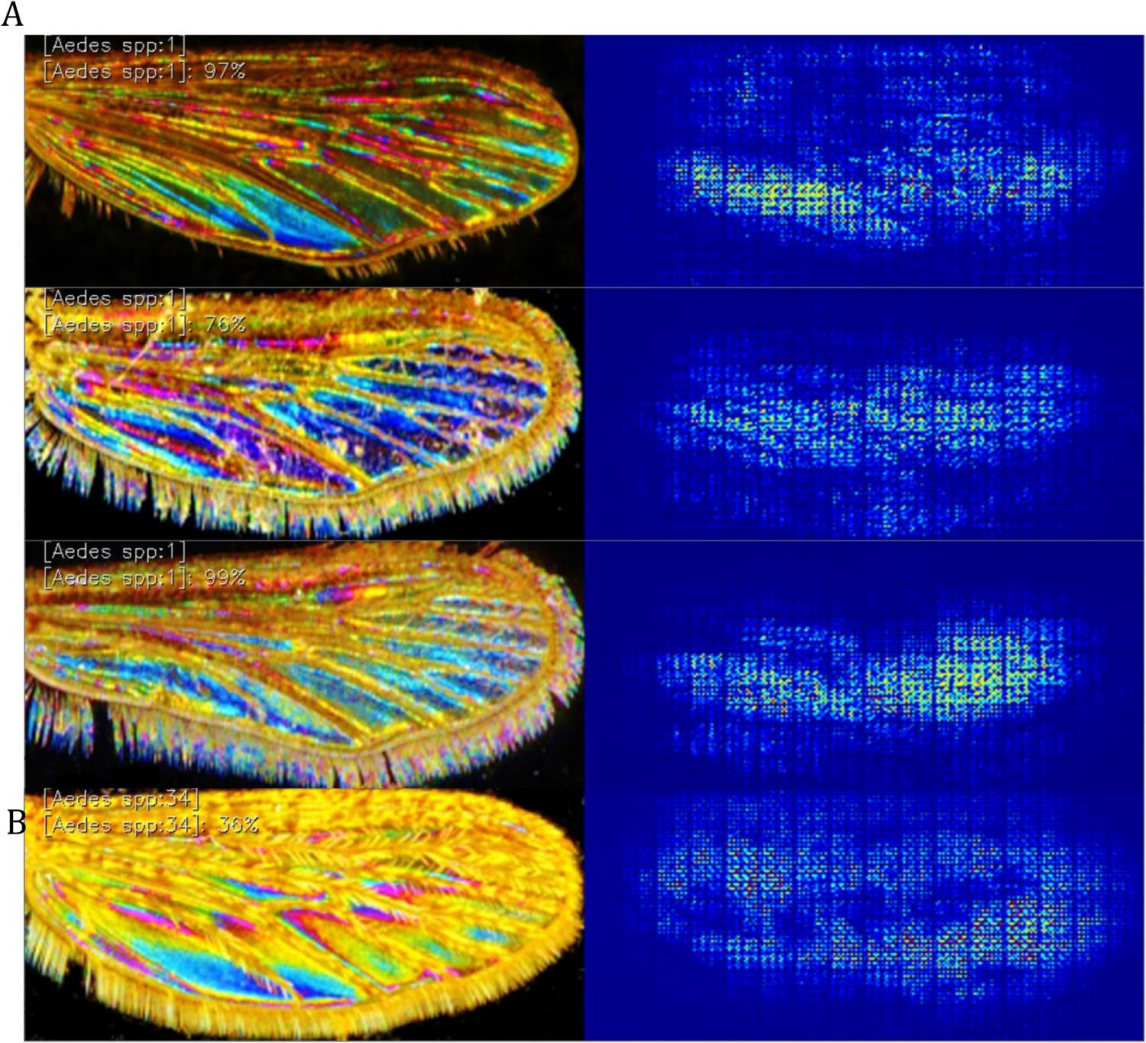
Activation maps for *Aedes* with a high confidence value (**A**) and low confidence value (**B**) on the prediction. Warm colors (yellow and red) of the activation point to wing regions where local features are essential for the decision.

Besides misclassified pictures, we generated activations maps pointing to which image region the architectures use, according to the feature extraction layers of the DCNN (Fig. 5).

These activation maps illustrate that the membrane and the vein structure are not targeted by the DCNN and that features activated concentrate in the lower part of the picture, particularly on the color pattern, when learning was successful Fig. 5 and high accuracy in the classification (Accuracy > 76%), while when classification accuracy is low (30%), features extracted spread all over the wing. In all cases, neither the wing shape nor the venation are features targeted by DCNN.

## Discussion

With robust vector surveillance, emerging vector-borne disease threats can be addressed. During an entomological survey, most routine identification involves skilled personnel that use diagnostic criteria, like the size, shape, or texture of specimens or the presence or absence of certain key features. Since the morphological identifications of medically important *Aedes* species is complex and meticulous, requiring highly skilled specialists, an alternative is to use computer vision that, like mosquito taxonomists, relies on visual (morphological) characters to assign a definitive taxon name. A framework based on a deep neural network was developed to extract mosquito anatomy from images. The process was tested on 23 Culicidae belonging to the *Aedes*, *Anopheles*, and *Culex* Genus^9^, but only 3 *Aedes* species (*Ae. aegypti*, *Ae. infirmatus*, and *Ae. taeniorhynchus)*. Nevertheless, manipulating mosquitoes to acquire and extract morphological characters is complicated and damages specimens. A real-time pipeline was set but has only been tested for discrimination of *Ae. aegypti* and *Ae. albopictus*^15^. Finally, deep convolutional network approaches using whole-body pictures of field-caught mosquitoes have shown promise, demonstrating astonishing accuracy, even classifying unknown/unidentified species before further identification^10^. Nevertheless, we can anticipate that morphology-based methods might suffer from the same limitation encountered by taxonomists for cryptic species/subspecies delineations, for *Aedes* species belonging to the Neomelanoconion identification whose require examination of internal organs, and as well as the misidentification of damaged specimens. Besides the mentioned methods, some characteristics like flight tone and wing beat, which play a role in mosquito reproduction^38–40^, were used for the automated identification assay of *Ae. albopictus* and *Ae. triseriatus*^39,40^, and more recent development permits an “at a distance” discrimination between *Ae. albopictus*, *Ae. aegypti* and *Culex* sp*.*^41,42^. Here, we prove the classification accuracy with WIPs is above 75% for 8 of the ten species investigated in our study.

Morphometry analysis of insects’ wings has demonstrated interest in vector mosquitoes and sibling or cryptic species^43–45^ identification. Nevertheless, this requires intact wings and extensive preprocessing to mount them without deformation. All that severely impedes its application for entomological survey purposes, but it is still attractive in taxonomic and populational studies. Our methodology would promise that *Aedes* species identification can be accurately performed with minimal training, cost, and time. In addition, wings are relatively robust insect organs that limit the risks of damaging them. In addition, they can be easily stored at room temperature and be carried with minimal caution.

Alternative methodologies not relying on morphological characteristics were implemented. These alternatives are DNA barcoding and protein profiling by MALDI-TOF, but they can also involve the wing beat features. MALDI-TOF has been applied to a restricted number of *Aedes* species at the egg stage, *i.e., Ae. albopictus*, *Ae. atropalpus*, *Ae. cretinus*, *Ae. geniculatus*, *Ae. koreicus*, *Ae. phoeniciae*, and *Ae. triseriatus,* 4 have medical importance^46,47^. It was also set up to identify the second larval stage to pupae of *Ae. albopictus* and *Ae. aegypti*^48^ and on the fourth instar larvae^49^ but with higher accuracy for late larval instar^50^. Its efficiency for field-trapped or colony-reared insect adults is limited. It includes species having (*Ae. albopictus, Ae. vigilax, Ae. vexans, Ae. notoscriptus, Ae. polynesiensis*) or not (*Ae. fowleri,* and *Ae. dufouri*) a medical importance^51–55^. However, the interoperability of the MALDI-TOF methodology requires a standardized procedure in the conservation of samples and the choice of the mosquito body part of adult specimens^56^. For MS identification, standardization of procedures for preparation and reproducibility between instruments and homemade databases will be desirable^57–60^. Contrary to MALDI-TOF or DNA-barcoding methodologies, our method is not designed to identify *Aedes* at larval stages. Nevertheless, a survey on adult mosquitoes is an alternative to larval surveys because it addresses the vector life stage responsible for transmission and thus adds information on vector density^61^. Infrared spectroscopy (NIR and MIR) can detect changes in the mosquito cuticle by quantifying light absorbed^62^. The discriminative capability of such methodology at the species level has been investigated on a restricted number of *Aedes* species having a medical interest (*Aedes aegypti*, *Ae. albopictus*, *Ae. japonicus*, and *Ae. triseriatus)*^63^*.* The main interest of this methodology relies on its capacity to grade the age of natural *Anopheles* spp. populations when coupled with a Deep learning approach^64^. DNA barcoding is also an alternative to morphological identification methods. A quest for “*Aedes*” as a keyword in the barcode of Life system (BOLD) database indicates 27,458 records with sequences representative of 328 species (http://v4.boldsystems.org/ accessed on 21 of July 2022)^65^. During proactive surveillance of invasive and/or medically and veterinary important species, genetic identification by PCR is costly, destructive, and time-consuming^66^, a limitation that does not carry our method. Prompt and correct identification of exogenous species is required to monitor entry points under the International Health Regulations (IHR) to limit the dispersal and establishment of new vector species. In this case, even entomologists experienced in identifying local mosquito fauna may experience difficulty accurately identifying damaged specimens. We provide evidence that WIPs analysis coupled with Deep Learning for discriminative identification competency to accurately classify *Aedes* species at various taxonomic levels (genus, subgenus, and species). The competency to accurately classify *Aedes* samples at the subgenus level is of interest to medical entomologists and public health services since a restricted number of *Aedes* subgenus encompass species of medical interest. In this study, among *Aedes* species accurately classified (Accuracy > 75%), four are of medical interest (*Ae. albopictus*, *Ae. aegypti*, *Ae. luteocephalus*, and *Ae. polynesiensis*). Therefore, we can envision that a database of WIPs encompassing proven *Aedes* vectors at a global or regional scale would allow remote territories to address the vector status of a specimen.

Increasing the area of vector surveillance is of high interest, even at a continental scale. Community science relying on the participation of non-expert people was therefore applied for mosquitoes^67,68^. Unfortunately, the quality of entomological data can be poor in terms of identification accuracy. Deep learning approaches have offered opportunities to overcome this limitation and have been developed to follow *Ae. albopictus* in Europe, from pictures taken by citizen^11^. However, this methodology wasn’t tested in high *Aedes* species diversity areas. Unfortunately, our method, as it stands, cannot be translated for this purpose because of the cost associated with microscopy and the need for high-quality pictures. Nevertheless, because wings are tiny and light, they can quickly be sent for identification purposes with this methodology. A global comparison between the various methods developed for Aedes identification is given in Table 5.

**Table 5:** Synthetic view of advantages and limitations of some identification methodologies for medically important arthropods in the framework of pro-active or large-scale entomological surveys.

Methods	Conservation procedure for identification	Technical cost*	Computational cost	Effort	Sample destruction	Currently available for *Aedes* sp.
DNA Barcoding	− 20 °C	High	Low	High	Yes	**328/**900 *Aedes* sp.
Protein Profiling (MALDI-TOF)	4 °C at best	High	Low	Low	Yes/part	**7**/900 *Aedes* sp.
Dichotomous keys	RT	High	Low	High	No/yes$	Yes regional
Near Infrared spectroscopy	− 20 °C	Medium	Low	Low	No	**4**/900
WIPs + Deep Learning (ours)	RT	Low	Medium	Low	No	**10**/900 *Aedes* sp.

Number of Aedes species tested.

*The technical cost includes sample preparation (DNA, protein extraction, slides, etc.) and expenses linked to the need for a skilled professional and the presence of dedicated infrastructure for running experiments.

Current global vector surveillance systems are unstandardized, burdensome on public health systems, and threatened by the worldwide scarcity of entomologists. The Deep learning analysis of *Aedes* WIPs resulting in robust classification could empower non-experts to identify disease vectors accurately and rapidly in the field. This is of interest in areas where large-scale and/or proactive surveys must be performed, like the Pacific area, where more than 20,000 islands must be surveyed^56,69^. This situation would make exogenous species introduction unnoticed. Nevertheless, the methods need now to be further investigated by evaluating, even qualitatively, whether the proposed approach could be usable in real-life scenarios regarding several important criteria: cost (including infrastructure, material, training, and cost associated with technically skilled personnel), computational resources, analyzing time, sample destructiveness, diversity, and taxonomic level (species, sibling species, cryptic species, sub-species) of the classification. All these will increase vector surveillance's programmatic capability and capacity with minimal training and cost.

### Supplementary Information

Supplementary Information 1.

Supplementary Information 2.

## Data Availability

The whole database, including *Aedes* species WIPS, is available on https://figshare.com/s/597f4e2f2d8206e09603, and the code on https://github.com/marcensea/diptera-wips.git.
