# Supplementary material for: Wing Interferential Patterns (WIPs) and machine learning for the classification of some Aedes species of medical interest
Source: Sci Rep. 2023 Oct 17;13:17628. doi: 10.1038/s41598-023-44945-3 (PMC10582169; doi:10.1038/s41598-023-44945-3)
Supplement: Supplementary file 2 — Supplementary Information 2. [file 41598_2023_44945_MOESM2_ESM.docx]

Supplementary data S1: Identity of species included in the database for training and validation of the Aedes classification process.
